# Genome-Wide Profiling of Structural Genomic Variations in Korean HapMap Individuals

**DOI:** 10.1371/journal.pone.0011417

**Published:** 2010-07-02

**Authors:** Joon Seol Bae, Hyun Sub Cheong, Byung Lae Park, Lyoung Hyo Kim, Chang Soo Han, Tae Joon Park, Jason Yongha Kim, Charisse Flerida A. Pasaje, Jin Sol Lee, Hyoung Doo Shin

**Affiliations:** 1 Laboratory of Genomic Diversity, Department of Life Science, Sogang University, Seoul, Republic of Korea; 2 Department of Genetic Epidemiology, SNP Genetics, Inc., Seoul, Republic of Korea; University College Dublin, Ireland

## Abstract

**Background:**

Structural genomic variation study, along with microarray technology development has provided many genomic resources related with architecture of human genome, and led to the fact that human genome structure is a lot more complicated than previously thought.

**Methodology/Principal Findings:**

In the case of International HapMap Project, Epstein-Barr various immortalized cell lines were preferably used over blood in order to get a larger number of genomic DNA. However, genomic aberration stemming from immortalization process, biased representation of the donor tissue, and culture process may influence the accuracy of SNP genotypes. In order to identify chromosome aberrations including loss of heterozygosity (LOH), large-scale and small-scale copy number variations, we used Illumina HumanHap500 BeadChip (555,352 markers) on Korean HapMap individuals (*n* = 90) to obtain Log R ratio and B allele frequency information, and then utilized the data with various programs including Illumina ChromoZone, cnvParition and PennCNV. As a result, we identified 28 LOHs (>3 mb) and 35 large-scale CNVs (>1 mb), with 4 samples having completely duplicated chromosome. In addition, after checking the sample quality (standard deviation of log R ratio <0.30), we selected 79 samples and used both signal intensity and B allele frequency simultaneously for identification of small-scale CNVs (<1 mb) to discover 4,989 small-scale CNVs. Identified CNVs in this study were successfully validated using visual examination of the genoplot images, overlapping analysis with previously reported CNVs in DGV, and quantitative PCR.

**Conclusion/Significance:**

In this study, we describe the result of the identified chromosome aberrations in Korean HapMap individuals, and expect that these findings will provide more meaningful information on the human genome.

## Introduction

A study involving analysis on the structure of a genome is very important because it can describe individual genetic differences and discover genetic factors that can affect phenotypes such as disease susceptibility. With the recent discovery of copy number variation [1], [2], scientists have realized that individual genetic differences are a lot more varied and complicated than previously expected, and studies on how these variations affect the risk of disease have been ongoing [2], [3], [4], [5]. Recently, with the advancement of next generation sequencer (NGS) and the fast development of computational analysis technologies such as massive paired-end mapping and Pindel [6], [7], [8], CNV research has shown big progress. Also, a very accurate high-resolution mapping of human genomic variations is expected to be completed when the 1000 Genomes Project (www.1000genomes.org) is finished. In the case of microarray used in CNV study, high-density chip is being developed continually, and consequently, the numbers of CNVs discovered are increasing [9]. Although the use of NGS provides exact CNV breakpoint and abundant genomic resources, it is still very expensive to use and analysis of huge raw data is very complicated. On the other hand, with microarray, genomic resources from a huge number of individuals can be attained at a relatively cheap price, which also benefits from various programs developed for analysis and are currently in use. Chromosome aberration is defined by a change in the chromosome structure or number including duplication, loss of heterozygosity (LOH), inversion and translocation. Chromosome aberration is usually found in cancer cells and is reported to be related with many other diseases [10], [11], [12].

The goal of International HapMap Project is to discover and provide all the variations in human genome, so as to promote the genetic study of complex diseases. Although Japan (JPN) and China (CHB) population are often combined into a single Asian sample in International HapMap Project, a recent study reported on the diverse characteristics between the two populations based on autosomal and Y-chromosomal markers [13]. He *et al.* claimed that conclusions derived from studies conducted on the combined Asian (ASN) population, JPN and CHB should be interpreted with caution. In addition, the authors discussed that the Korean population is distinct from an Asian HapMap sample [13]. Despite having different regions, Yamaguchi-Kabata *et al* reported on a genetic differentiation observed in Japan [14]. Although Korea is geographically located between both China and Japan, Korean population does not necessarily share the same genetic background with either population. Korean government initiated the Korean HapMap project with goals of finding genetic variation unique to Koreans and genomic studying of Koreans. With international HapMap Project being such a large scale, a lot of genomic DNAs are required, and in order to meet this requirement, Epstein-Barr virus immortalized lymphocytes [lymphoblast cell line (LCLs)] are used instead of blood, which could result in genomic aberration [15]. Because of this, Redon *et al.,* investigated chromosome aberration on cell lines using CGH array and SNP array [2] before undergoing human CNV discovery research. However, such aberrations may lead to wrong SNP genotype call which is a problem for the present project in achieving its goal. Also, when blood-extracted genomic DNA and LCL-extracted samples were comparatively analyzed for genomic alteration, similar variations were found in blood samples but more aberrations were observed in LCLs [15]. These results suggest that studies using LCLs sample need to undergo advance investigation for chromosome aberration. Especially in stem cell studies wherein chromosome aberrations need to be discovered fast and conveniently before being applied clinically [15].

In this study, we analyzed chromosome aberrations on Korean HapMap individuals using high-density SNP array, and we expected that the results would provide meaningful information for understanding human genome.

## Results

**Figure 1:**
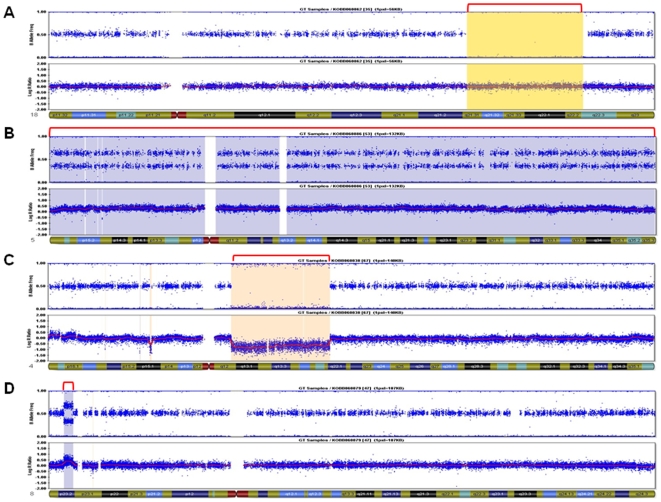
Visualization of identified loss of heterozygosity and large-scale copy number variation (>1 mb). A. Plot of B allele frequency and log R ratio on chromosome 18 in sample KOBB060862. Each panel includes plot of B allele frequency (upper) and log R ratio (lower). The plot of B allele frequency shows genotype for AA, AB and BB, and the plot of log R ratio indicates signal intensity for each marker which shows copy number changes. The red line in the plot shows mean of log R ratio over 1 mb sliding window. A region representing copy-neutral loss of heterozygosity (LOH) appears in the plot of log R ratio (yellow colored box). B. Visualization of duplication on entire chromosome 5 in sample KOBB060886. The plot of B allele frequency shows four lines including top and bottom lines. In addition, the signal intensities of markers are increased in all position. C. Visualization of duplication on chromosome 8 in sample KOBB060879. The Plot of B allele frequency and log R ratio on chr8: 3674807–5938053 (size: 2.3 mb) indicates where occurred a duplication variation. D. Visualization of deletion on chromosome 4 in sample KOBB060838. The region (chr4:57782628–88983457) shows LOH and decreasing of intensity (bisque colored box).

**Figure 2:**
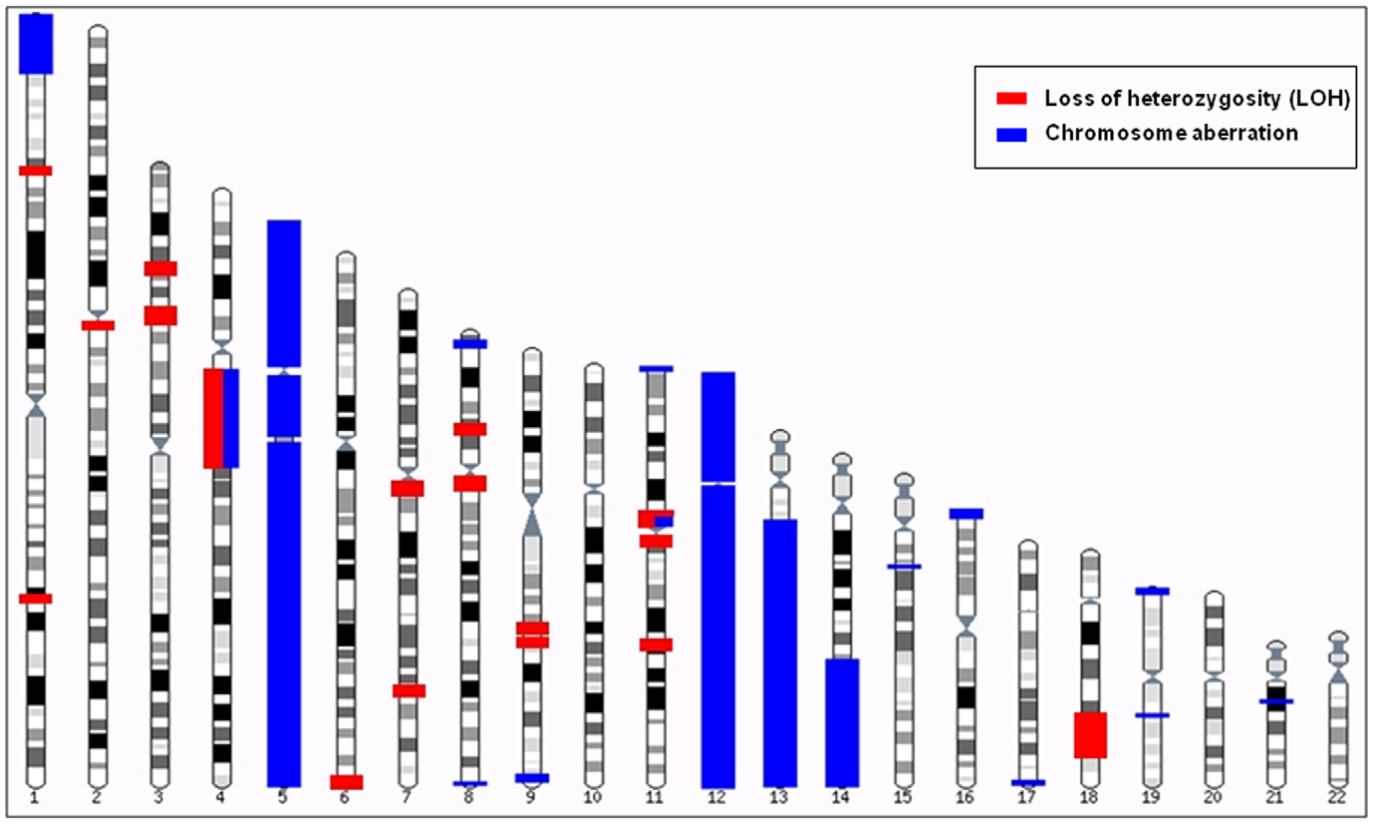
Map of loss of heterozygosity and chromosomal aberrations due to cell culture artifacts in HapMap individuals (*n* = 90).

In this study, we used Illumina HumanHap500 BeadChip (555,352 markers) and three types of program to identify chromosome aberrations including loss of heterozygosity (LOH), large-scale, and small-scale copy number variations in Korean HapMap individuals. In order to identify loss of heterozygosity (LOH), we used ChromoZone program on 90 samples and found 28 LOH regions from 22 samples with a total length of 141.7 mb and an average length of 1.6 mb. Among the 28 regions, only chr4:58000000–89000000 region from KOBB060838 sample was a copy-loss LOH, whereas the others were copy-neutral LOH (cnLOH) (Table 1). We used cnvPartition, a program often used for large-scale aberration discovery to identify large-scale copy number variations including duplication and deletion over 1 mb, and found 35 aberrations from 17 samples. From the 35 chromosome aberrations, KOBB060876, KOBB060886, KOBB060889 and KOBB060890 showed complete duplication of chromosome 12, and KOBB060886 showed complete duplication of chromosome 5 (Table S1). Chromosome 12 repeatedly showed an entire duplication pattern, which was quite peculiar. Figure 1 displays identified loss of heterozygosity and large-scale copy number variation (>1 mb) plotted against B allele frequency (allelic intensity) and log R ratio (signal intensity). Heterozygosity disappeared in cnLOH without change in copy number (Figure 1A). Entire duplication that appeared in 4 samples showed heterozygosity from plot of B allele frequency split into two lines, which increased all the signal intensity (Figure 1B). KOBB060838 sample had a large-scale deletion (Size: 31.0 mb) in chr4:58000000–89000000 region. Also, in the plot of B allele frequency of the sample, LOH was found in the same region which decreased all signal intensity (Figure 1C). Figure 1D shows large-scale duplication in chr8:3674807–5938053 (Size: 2.3 mb) of KOBB060879 sample, with split of heterozygosity clearly displayed in plot of B allele frequency and increase of signal intensity. Figure 2 displays the map of chromosome aberration including LOH, large-scale deletion, and duplication found in this study.

**Table 1:** Regions of identified loss of heterozygosity (>3 Mb) using Illumina ChromoZone (*n* = 90).

Sample ID	Chromosome	Position (Start)†	Position (Start)†	Size (Mb)
KOBB060804	2	94,000,000	97,000,000	3.0
KOBB060805	7	126,100,000	130,100,000	4.0
KOBB060806	2	94,000,000	97,000,000	3.0
KOBB060807	11	46,500,000	50,500,000	4.0
KOBB060808	7	61,100,000	66,100,000	5.0
KOBB060810	1	49,000,000	52,000,000	3.0
KOBB060821	3	32,000,000	36,000,000	4.0
KOBB060825	8	46,500,000	49,700,000	3.2
KOBB060829	11	47,300,000	50,500,000	3.2
*KOBB060838	4	58,000,000	89,000,000	31.0
KOBB060839	3	49,000,000	52,000,000	3.0
	9	88,200,000	91,600,000	3.4
	9	92,400,000	95,800,000	3.4
KOBB060853	3	47,000,000	50,000,000	3.0
KOBB060858	8	46,500,000	49,700,000	3.2
KOBB060860	11	46,000,000	51,300,000	5.3
KOBB060862	6	167,300,000	170,900,000	3.6
	8	46,500,000	52,100,000	5.6
	18	52,600,000	67,200,000	14.6
KOBB060863	11	47,800,000	51,300,000	3.5
	11	54,100,000	57,600,000	3.5
KOBB060864	1	185,800,000	188,800,000	3.0
KOBB060874	3	47,000,000	50,000,000	3.0
KOBB060880	8	30,500,000	34,500,000	4.0
	11	48,100,000	51,300,000	3.2
KOBB060881	3	46,000,000	52,000,000	6.0
KOBB060882	11	46,900,000	50,400,000	3.5
KOBB060888	11	87,400,000	90,900,000	3.5

* Copy-loss LOH.

† The version of human reference genome: NCBI build 36/hg18.

**Figure 3:**
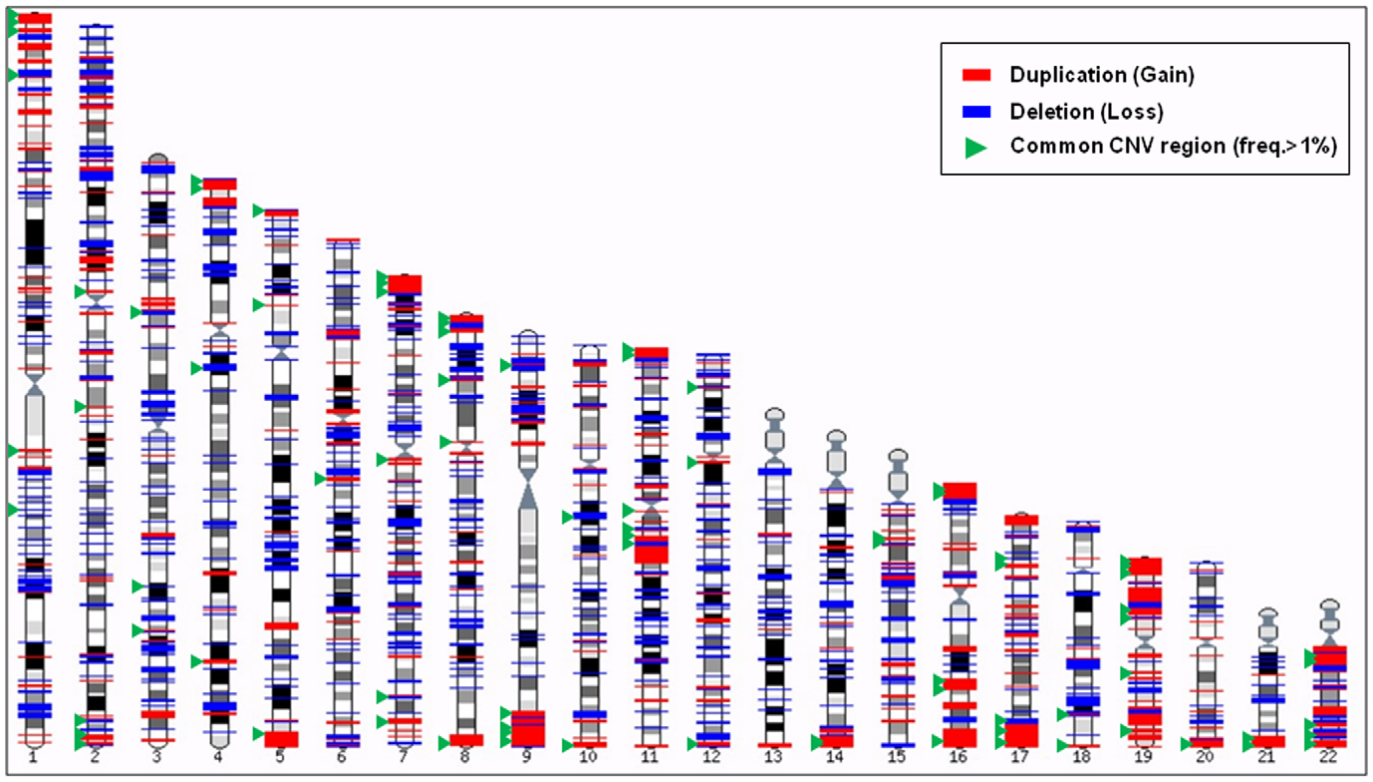
Map of identified small-scale copy number variations in HapMap individuals (*n* = 79).

To find small-scale copy number variations, we simultaneously used both signal intensity and B allele frequency. In addition, we selected 79 samples after quality check and removed identified chromosome aberration regions for detection of reliable small-scale CNVs. As a result, we identified a total of 4,989 individual small-scale CNVs and 1,324 small-scale CNV regions (Table 2; Table S2). Included in our findings were a total of 123 common small-scale CNVRs (frequency >1.0%) and 2,493 genes (Table S3). Figure S1 shows the size distribution of identified CNVs and aggregated CNV regions in this study. Most of the identified CNVs and CNVRs were distributed within 1–50 kb in range. Figure 3 displays genetic map of the identified CNVs with one hundred and twenty three common small-scale CNVs represented by green triangles. Identified small-scale CNVs in this study were compared with previously reported CNVs from database of genomic variants (http://projects.tcag.ca/variation/), and were found to overlap with 3,741 of 4,989 small-scale CNVs (75.0%). After running 50% reciprocal overlapping analysis on 3,741 CNVs using alternative scan script in pennCNV, we found that 1,026 CNVs (27.4%) overlapped with CNVs in DGV. In order to validate the identified CNVs, we performed quantitative PCR for four randomly selected CNVs. Quantitative measurement values of all samples in four regions (chr4:162083343–162146977 and chr12:11411688–11434269 for duplication, chr1:147305744–147427061 and chr4:10006425–1000925 for deletion) matched the expected copy number through estimation by visual examination (Figure S2; Figure S3).

**Table 2:** Identified small-scale copy number variations using PennCNV in HapMap individuals (*n* = 79).

Parameters	Count
CNV	
- Total number	4,989
- Avg. number of CNVs per sample	63.2
- Avg. size of CNVs (kb)	70.6
- Median size of CNVs (kb)	39.9
- Number of gain	1,960
- Number of loss	3,029
- Ratio (Loss/Gain)	1.5
CNV region	
- Total number	1,324
- Avg. number of CNVs per sample	16.8
- Avg. size of CNVs (kb)	80.3
- Median size of CNVs (kb)	36.0
- Number of common CNVs (freq.>1%)	123
- Number of common CNVs (freq.>2.5%)	40
- Number of common CNVs (freq.>5%)	16
- Genes	2,493

## Discussion

Chromosome aberration can lead to a variety of genetic disorders such as Down syndrome, Jacobsen syndrome, Hodgkin disease and Turner syndrome [16], [17], [18]. Such large-scale aberrations have been usually analyzed using low resolution cytogenetic method. With recent advancements on SNP array technology, it is now possible to detect previously elusive genetic variations with high resolution. Originally, SNP array was developed to obtain individual SNP genotype, but now, it has also been considered as a tool for analyzing extended homozygosity and copy number change [15], [19]. This is because SNP array provides not only signal intensity for copy number but also B allele frequency, which is required for detecting LOH. We used Illumina HumanHap500 BeadChip (555,352 markers) on Korean HapMap individuals to investigate genome-wide profiling of structural genomic variations. As a result, we found 28 LOH regions from 22 samples, and 33 large-scale aberration including duplication and deletions from 19 samples. We also found 4,989 small-scale copy number variations with sizes ranging between 1 kb and 1 mb. One unusual finding was the fact that LOHs with sizes larger than 3 mb were mostly copy-neutral LOH (cnLOH). The loss of heterozygosity in cnLOH occurs without copy number change. In addition, cnLOH has been known to be developed by mitotic recombination or deletion and reduplication, and in fact, a previous study reported that it can affect the risk of tumorigenesis of *MUTHY*-associated polyposis (MAP) [20]. However, the underlying mechanisms of how cnLOH affects complex diseases are yet to be discovered.

Large-scale deletion and duplication is rarely found in healthy individuals. Simon-Sanchez *et al*. found 11 duplications and 2 deletions by blood sample analyses (*n* = 275) [15]. Except for one individual, the sizes of the duplications were smaller than 2.6 mb. On the other hand, 11 variations not initially detected in blood samples were found using LCLs analyses, with 4 variations occurring on an entire chromosome and other variations detected with larger sizes. In the present study, we were also able to discover variations from LCLs with larger sizes, such as 5 entire chromosome duplication, and similar to the findings of Simon-Sanchez *et al*, we were able to detect 10 variations with sizes larger than 3 mb. Some of the factors as to why such aberrations occur more in LCLs are suggested to be faulty use of donor tissue, the artifact of EBV immortalization process, creation during culture, etc [15]. Variations occurred using LCLs do not affect SNP genotype with huge samples, but may influence the accuracy of SNP genotype taking into account small sample size. Therefore, detecting abnormal individuals having chromosome aberration regions before SNP genotyping would be a good method to increase accuracy of SNP genotype. The present study took these problems into consideration and subsequently got rid of samples with aberrations larger than 1 mb. With these samples, we identified small-scale CNVs, and then removed abnormal regions from identified small-scale CNVs. As a result, we found 4,989 small-scale CNVs which overlapped at 75% when compared with previously found small-scale CNVs in Database of Genomic Variants (DGV). After comparing our results with two previous studies [21], [22] that discovered structural variants from Koreans by whole-genome sequencing, 44.9% (106/236 variants) [21] and 22.4% (820/3,662 variants) [22] of our CNVs appeared to overlap. The previous studies ran high-resolution scanning on one human sample, and are very significant in that it suggested the direction of future CNV research. As to why overlapping rate between the two papers and our CNVs was not high, we considered the differences in samples and platforms used. The ideal study method would be to use NGS (Next Generation Sequencer) on CNV studies, but obtaining genomic resource from more than a thousand samples by NGS would require an astronomical amount of fund. Since genomic resources of many samples are required in genetic epidemiological study, chip-based strategy is still going to be used as a main study tool. After comparing our identified CNVs and segmental duplications (SDs), we found that many CNVs were located in SDs region (43.5%). This observation is similar to the results of other studies [2], [23], [24] wherein CNVs discovered were closely related with segmental duplication (SD). Also, result from our gene ontology (GO) analysis showed that cell adhesion displayed similar distributions from previous studies. However, sensory perception of smell and neurophysiological processes did not have a highly enriched region in the GO category (Table S4).

In our study, we suggest a faster and more accurate method that is, the detection of chromosome aberrations of LCLs using advance studies and increase accuracy of acquired genomic resource information. Previously, array CGH and SNP array were usually used to detect chromosome aberration. However, a new method that uses Genome Viewer of BeadStudio software of Illumina HumanHap300 BeadChip (317K) to find large-scale CNVs including cnLOH was recently introduced [15]. Despite the accuracy and efficiency of this method, it requires visual examination from the user which may take longer time to analyze the data and may result to decreased efficiency. Based on these studies other programs, some of which have been utilized by the present study, that can automatically detect various chromosome aberrations, to suggest a fast and accurate method of discovering chromosome aberration have been developed. We expect this method will help find abnormal chromosomal regions rapidly and accurately for studies using stem cells or LCLs.

In summary, we genotyped Korean HapMap individuals using Illumina HumanHap500 BeadChip (555K), and used three programs to identify cnLOH, large-scale CNV and small-scale CNV. Findings from this study can be used for faster and more accurate detection of chromosome aberration, and the newly found genomic alterations will serve as important materials for understanding human genomic structure.

## Materials and Methods

### Subject collection and whole-genome SNP genotyping

All individuals included in this study were of Korean ethnic origin. The samples were obtained from Korean HapMap project (*n* = 90) which included 90 unrelated healthy Korean individuals (40–69 years). All genomic DNA of EB virus-transformed lymphoblastoid cell lines (LCLs) were collected from the National Biobank of Korea in Korean National Institute of Health as described in previous reports [25], [26]. All donated samples for genetic tests after signing the informed consent form that was approved by the relevant institutional review board (The Korean HapMap Project Consortium). We used the Illumina HumanHap500 BeadChip containing 555,352 markers (Illumina, Inc., USA) to obtain log R ratio (signal intensity) and B allele frequency (allelic frequency). Approximately 750 ng of genomic DNA, which was extracted from Epstein-Barr virus immortalized cell lines, was used to genotype each sample. The assay procedure has been described in our previous study [27]. Raw intensity and allelic data were normalized through five processes (outlier removal, translation correction, rotational correction, shear correction and scaling correction) using Illumina BeadStudio 3.2 software [19]. Also, in this study, a default cluster provided by Illumina encountered a problem where the value was different depending on ethnicity and experiment time, so we normalized all markers by automatic clustering. For an accurate CNV identification, we excluded samples with LRR_SD (standard deviation of log R ratio) value over 0.30 or samples with unusually high number of identified CNVs. The overall SNP genotyping call rate was 99.69%, which indicated that high-quality data was extracted for this study.

### Identification of chromosome aberration including loss of heterozygosity (>3 mb) and large-scale copy number variation (>1 mb)

In order to identify chromosome aberrations including loss of heterozygosity (>3 mb) and large-scale copy number variation (>1 mb), we used ChromoZone (Illumina, USA) and cnvPartition (Illumina, USA) programs. ChromoZone program was used to identify loss of heterozygosity (LOH) under default condition (confidence threshold: 35, detect extended homozygosity: true, include sex chromosomes: false, minimum homozygous region size: 5000000, mininum probe count: 3). Although we cannot identify large-scale copy number variations following this program because it only uses B allele frequency metric, still it is optimized to detect B allele frequency change (Illumina user bulletin: Pub. No. 970-2007-007). After running the program to detect LOH, outputs with Heterozygosity Sparse (Het. Sparse) were selected and analyzed. cnvPartition (ver. 1.2.1) was used to identify large-scale duplications and deletions under default condition (none of threshold). This program, unlike ChromoZone, is optimized to analyze intensity information, and therefore is a good tool in carrying out large-scale copy number estimations (Illumina user bulletin: Pub. No. 970-2007-007). For the analysis condition of the program, number of probe was adjusted to 10 SNPs and everything else was set to default for the analysis.

### Sample selection and identification of small-scale copy number variation (<1 mb)

We used high quality samples to identify reliable small-scale copy number variations with call rate >99.0%, number of small-scale CNVs <177 [(Average number of identified small-scale CNVs per sample (87.6) +1 standard deviation (89.3)], and standard deviation (SD) of log R ratio (LRR) <0.30 being the criteria. However, we did not use the wave-adjust function for identifying CNV in pennCNV because the selected samples had good waviness factor values (WF value) (average of WF ± standard deviation  = 0.01±0.03). In order to identify authentic small-scale CNVs, we used multiple factors including log R ratio (LRR), B allele frequency (BAF), marker distance, and population frequency of the B allele. After these parameters were extracted using Illumina BeadStudio 3.2 (Illumina, USA), individual small-scale CNVs were identified by pennCNV [28], [29]. We downloaded all scripts and library files including hhall.hg18.pfb from the website (http://www.openbioinformatics.org/penncnv), and we performed the identification of small-scale CNVs by the described procedure in Kai *et al* [28].

### Validation by quantitative PCR

To validate the existence of both the CNV region, we performed TaqMan real-time PCR on an ABI Prism 7900 sequence detection system (Applied Biosystems, Foster City, CA) for four randomly selected CNVs among the identified CNVs in this study. Specific probes were generated by Primer Express 2.0 (Table S5). The *RNaseP* gene (part number: 4403328) was co-amplified with the marker which was then used as an internal standard. Amplification reactions (10 ul) were carried out using 10 ng of template DNA, 1× TaqMan Universal Master Mix buffer (Applied Biosystems, Foster City, CA), 900 nM of each primer, and 250 nM of each fluorogenic probe. Thermal cycling was initiated with 2 min incubation at 50°C, followed by a first denaturation step of 10 min at 95°C, and then 40 cycles of 15 sec at 95°C and 1 min at 60°C. Three replicate reactions were performed for similar primer pairs and each copy number of individuals was calculated by Copy Caller v1.0 (Applied Biosystems, Foster City, CA) using comparative C_T_ method.

### Overlapping analysis and gene ontology analysis

For overlap analysis of both our identified CNVs and previously reported CNVs in DGV, we used “scan_region.pl” script provided by penncnv algorithm. Second, 50% reciprocal overlapping analysis was performed by “scan_region.pl -minquerydbratio.5” script (http://www.openbioinformatics.org/gengen/tutorial_scan_region.html). Gene ontology (GO) analysis can provide insight into the functional enrichment of CNVs. For this reason, we performed GO analysis using GOstat (http://gostat.wehi.edu.au) provided by Tim Beiβbarth [30], [31].

### CNV mapping

We used the karyoview of Ensemble for drawing the maps of the identified genomic variations including CNVs (http://apr2006.archive.ensembl.org/Homo_sapiens/karyoview). The input data of each variation were prepared following this format; chromosome, start position, end position and status (optional). The input data of all variants were fixed to “paste file content” menu before the map was drawn.

## Supporting Information

Figure S1 Size distribution of identified copy number variations and aggregated copy number variation regions.
(0.03 MB DOC)

Figure S2 Quantitative validation result by qPCR in two selected deletion regions. A. Two-dimensional genoplot image representing allelic intensity (X-axis) and signal intensity (Y-axis) in rs11579261 probe within chr1:147305744-147427061. B. Two-dimensional genoplot image and the result of qPCR in rs4607209 probe within chr4:10006425-10009254. The estimated copy number value through estimation by visual examination matched quantitative measurement value by qPCR.
(0.28 MB DOC)

Figure S3 Quantitative validation result by qPCR in two selected duplication regions. A. Two-dimensional genoplot image and the result of qPCR in rs9308018 probe within chr4:162083343-162146977. B. Two-dimensional genoplot image and the result of qPCR in rs10845343 probe within chr12:11411688-11434269. Two CNVs representing duplication were successfully validated by qPCR.
(0.25 MB DOC)

Table S1 Regions of identified large-scale copy number variations (>1 Mb) using Illumina cnvPartition (n = 90).
(0.09 MB DOC)

Table S2 List of identified small-scale copy number variations in Korean HapMap individuals (n = 79).
(1.01 MB XLS)

Table S3 Result of aggregated small-scale copy number variation regions in Korean HapMap individuals (n = 79).
(0.26 MB XLS)

Table S4 Gene ontology categories significantly overrepresented in identified copy number variations.
(0.07 MB DOC)

Table S5 Primer sequences for quantitative PCR.
(0.02 MB XLS)
