# Supplementary material for: Genome-Wide Profiling of Structural Genomic Variations in Korean HapMap Individuals
Source: PLoS One. 2010 Jul 2;5(7):e11417. doi: 10.1371/journal.pone.0011417 (PMC2896390; doi:10.1371/journal.pone.0011417)
Supplement: Table S1 — Regions of identified large-scale copy number variations (>1 Mb) using Illumina cnvPartition (n = 90). (0.09 MB DOC) [file pone.0011417.s004.doc]

Table S1. Regions of identified large-scale copy number variations (>1Mb) using Illumina cnvPartition (n=90)

| Sample ID | Chromosome | Position (Start) ^†^ | Position (Start) ^†^ | Size (Mb) | Type of aberration |
| --- | --- | --- | --- | --- | --- |
| KOBB060803 | 11 | 48,283,349 | 51,382,454 | 3.1 | Deletion |
| KOBB060809 | 9 | 136,165,607 | 137,799,266 | 1.6 | Duplication |
|  | 11 | 275,936 | 1,464,088 | 1.2 | Duplication |
|  | 17 | 76,540,709 | 77,678,922 | 1.1 | Duplication |
|  | 19 | 489,531 | 2,200,477 | 1.7 | Duplication |
| KOBB060814 | 21 | 19,118,359 | 20,122,483 | 1.0 | Duplication |
| KOBB060818 | 1 | 5,107,112 | 19,541,157 | 4.0 | Duplication |
|  | 1 | 792,429 | 4,823,099 | 14.4 | Duplication |
|  | 9 | 136,088,711 | 138,303,776 | 2.2 | Duplication |
|  | 16 | 259,512 | 3,040,096 | 2.8 | Duplication |
|  | 19 | 243,082 | 2,091,009 | 1.8 | Duplication |
| KOBB060820 | 8 | 144,401,827 | 145,700,535 | 1.3 | Duplication |
|  | 9 | 136,277,119 | 137,799,266 | 1.5 | Duplication |
| KOBB060824 | 13 | 28,895,689 | 95,982,289 | 67.1 | Duplication |
|  | 13 | 96,317,612 | 102,787,731 | 6.5 | Duplication |
|  | 13 | 103,339,702 | 110,528,646 | 7.2 | Duplication |
|  | 13 | 110,824,624 | 114,121,252 | 3.3 | Duplication |
|  | 14 | 104,686,693 | 106,127,399 | 1.4 | Duplication |
| KOBB060836 | 11 | 50,288,640 | 51,303,111 | 1.0 | Deletion |
| KOBB060838 | 4 | 57,782,628 | 88,983,457 | 31.1 | Deletion |
| KOBB060839 | 12 | 50,450,056 | 132,389,146 | 81.9 | Deletion |
|  | 14 | 65,803,279 | 106,358,708 | 40.6 | Duplication |
| KOBB060855 | 15 | 29,271,979 | 30,300,468 | 1.0 | Duplication |
|  | 19 | 40,157,027 | 41,365,140 | 1.2 | Duplication |
| KOBB060868 | 9 | 136,211,379 | 137,799,266 | 1.6 | Duplication |
|  | 16 | 259,512 | 1,513,891 | 1.3 | Duplication |
| KOBB060876 | 12 | 64,079 | 34,711,193 | Entire chromosome | Duplication |
|  | 12 | 36,219,721 | 50,927,395 |  | Duplication |
|  | 12 | 51,061,301 | 69,158,125 |  | Duplication |
|  | 12 | 69,168,994 | 110,187,905 |  | Duplication |
|  | 12 | 112,016,201 | 132,389,146 |  | Duplication |
| KOBB060879 | 8 | 3,674,807 | 5,938,053 | 2.3 | Duplication |
| KOBB060885 | 9 | 136,303,288 | 137,799,266 | 1.5 | Duplication |
| KOBB060886 | 5 | 80,564 | 10,559,509 | Entire chromosome | Duplication |
|  | 5 | 10,746,687 | 14,379,955 |  | Duplication |
|  | 5 | 14,562,903 | 15,772,350 |  | Duplication |
|  | 5 | 15,774,360 | 46,384,240 |  | Duplication |
|  | 5 | 49,618,507 | 68,776,409 |  | Duplication |
|  | 5 | 70,715,382 | 180,623,543 |  | Duplication |
|  | 12 | 64,079 | 6,893,384 | Entire chromosome | Duplication |
|  | 12 | 7,255,709 | 34,711,193 |  | Duplication |
|  | 12 | 36,219,721 | 69,160,993 |  | Duplication |
|  | 12 | 69,168,994 | 132,389,146 |  | Duplication |
| KOBB060889 | 12 | 64,079 | 34,711,193 | Entire chromosome | Duplication |
|  | 12 | 36,219,721 | 56,156,422 |  | Duplication |
|  | 12 | 56,585,517 | 132,389,146 |  | Duplication |
| KOBB060890 | 12 | 64,079 | 34,711,193 | Entire chromosome | Duplication |
|  | 12 | 36,219,721 | 69,158,125 |  | Duplication |
|  | 12 | 69,168,994 | 132,389,146 |  | Duplication |

^†^ The version of human reference genome: NCBI build 36/hg18
