# Supplementary material for: Genome-Wide Profiling of Structural Genomic Variations in Korean HapMap Individuals
Source: PLoS One. 2010 Jul 2;5(7):e11417. doi: 10.1371/journal.pone.0011417 (PMC2896390; doi:10.1371/journal.pone.0011417)
Supplement: Table S4 — Gene ontology categories significantly overrepresented in identified copy number variations. (0.07 MB DOC) [file pone.0011417.s007.doc]

| Table S4. Gene ontology categories significantly overrepresented in identified copy number variations | | | | | |
| --- | --- | --- | --- | --- | --- |
| Group | GO id | Ontology | Count | Total | P-value |
| Included genes in all identifed CNVs | GO:0005515 | protein binding | 857 | 9005 | 6.50E-62 |
|  | GO:0005737 | cytoplasm | 720 | 7482 | 2.53E-51 |
|  | GO:0044464 | cell part | 1569 | 21746 | 4.62E-41 |
|  | GO:0044424 | intracellular part | 1050 | 12958 | 1.69E-39 |
|  | GO:0043231 | intracellular membrane-bound organelle | 759 | 8824 | 1.40E-33 |
|  | GO:0043227 | membrane-bound organelle | 759 | 8827 | 1.40E-33 |
|  | GO:0043229 | intracellular organelle | 865 | 10763 | 8.48E-28 |
|  | GO:0043226 | organelle | 865 | 10768 | 8.93E-28 |
|  | GO:0032502 | developmental process | 343 | 3347 | 7.53E-27 |
|  | GO:0065007 | biological regulation | 588 | 6731 | 5.16E-26 |
|  | GO:0005886 | plasma membrane | 375 | 3816 | 2.00E-25 |
|  | GO:0005622 | intracellular | 1106 | 14906 | 5.60E-24 |
|  | GO:0016020 | membrane | 781 | 9747 | 1.14E-23 |
|  | GO:0050789 | regulation of biological process | 531 | 6140 | 8.47E-22 |
|  | GO:0005624 | membrane fraction | 102 | 683 | 1.83E-21 |
|  | GO:0048856 | anatomical structure development | 221 | 2005 | 2.48E-21 |
|  | GO:0044425 | membrane part | 637 | 7726 | 2.64E-21 |
| Included genes in noble CNVs in Korean population | GO:0005515 | protein binding | 351 | 9005 | 8.26E-33 |
|  | GO:0016337 | cell-cell adhesion | 36 | 347 | 3.16E-21 |
|  | GO:0022610 | biological adhesion | 62 | 960 | 2.75E-16 |
|  | GO:0007155 | cell adhesion | 62 | 960 | 2.75E-16 |
|  | GO:0048856 | anatomical structure development | 102 | 2005 | 2.76E-16 |
|  | GO:0044464 | cell part | 598 | 21746 | 5.39E-16 |
|  | GO:0005624 | membrane fraction | 49 | 683 | 8.20E-16 |
|  | GO:0005886 | plasma membrane | 158 | 3816 | 6.77E-15 |
|  | GO:0005737 | cytoplasm | 259 | 7482 | 6.25E-14 |
|  | GO:0016020 | membrane | 315 | 9747 | 3.49E-13 |
|  | GO:0048731 | system development | 82 | 1605 | 4.02E-13 |
|  | GO:0007275 | multicellular organismal development | 105 | 2299 | 9.93E-13 |
|  | GO:0044425 | membrane part | 261 | 7726 | 1.06E-12 |
|  | GO:0032502 | developmental process | 138 | 3347 | 1.07E-12 |
|  | GO:0031224 | intrinsic to membrane | 231 | 6732 | 1.20E-11 |
|  | GO:0016021 | integral to membrane | 230 | 6700 | 1.25E-11 |
|  | GO:0000267 | cell fraction | 52 | 902 | 8.63E-11 |
|  | GO:0044424 | intracellular part | 383 | 12958 | 9.18E-11 |
